# Predictors of adherence to exercise interventions during and after cancer treatment: A systematic review

**DOI:** 10.1002/pon.4612

**Published:** 2018-01-26

**Authors:** H.L. Ormel, G.G.F. van der Schoot, W.J. Sluiter, M. Jalving, J.A. Gietema, A.M.E. Walenkamp

**Affiliations:** ^1^ Department of Medical Oncology, University Medical Center Groningen University of Groningen Groningen The Netherlands; ^2^ Department of Endocrinology, University Medical Center Groningen University of Groningen Groningen The Netherlands

**Keywords:** cancer, exercise, exercise intervention, neoplasms, oncology, patient compliance, patient dropouts, physical exercise, prediction of adherence, systematic review

## Abstract

**Objective:**

Exercise interventions benefit cancer patients. However, only low numbers of patients adhere to these interventions. This review aimed to identify predictors of exercise intervention adherence in patients with cancer, during and after multimodality cancer treatment.

**Methods:**

A literature search was performed using electronic databases (PubMed, Embase, and Cochrane) to identify relevant papers published before February 1, 2017. Papers reporting randomized controlled trials, conducted in adult cancer patients who participated in an exercise intervention during and/or after multimodality cancer treatment, and providing outcome of factors predicting exercise adherence were included. Papers were assessed for methodological quality by using the Physiotherapy Evidence Database scale.

**Results:**

The search identified 720 potentially relevant papers, of which 15 fulfilled the eligibility criteria. In these 15 studies, 2279 patients were included and 1383 of these patients were randomized to an exercise intervention. During cancer treatment, the factors predicting exercise adherence were as follows: location of the rehabilitation center, extensive exercise history, high motivation for exercise, and fewer exercise limitations. After cancer treatment, factors that predicted adherence were as follows: less extensive surgery, low alcohol consumption, high previous exercise adherence, family support, feedback by trainers, and knowledge and skills of exercise. Methodological quality of the included papers was rated “high”.

**Conclusions:**

The most prominent predictors of adherence to exercise interventions were location of the rehabilitation center, extensive exercise history, high motivation for exercise, and fewer exercise limitations. To increase the number of cancer patients who will benefit, these results should be considered into the development and implementation of future exercise interventions.

## 1 INTRODUCTION

Cancer affects millions of people worldwide, and in 2012, the reported incidence was 14.1 million.^1^ Earlier and more accurate cancer diagnosis in combination with better treatments have improved cancer survival.^2^, ^3^, ^4^ Over the last 2 decades, survival rates have increased significantly. In the United States alone, there were more than 14 million cancer survivors and these numbers are expected to increase up to an estimated 18 million in 2020.^2^, ^3^

Increasingly, depending on cancer type, stage, and (genetic) characteristics, patients receive multimodality cancer treatment, often including surgery, radiotherapy, and/or systemic treatment.^5^ Cancer treatment can result in deterioration of physical fitness, decreased muscle strength, fatigue, and a reduced quality of life.^6^, ^7^, ^8^ Cancer treatment can also result in inactivity and weight gain, as previously described in patients diagnosed with breast cancer, prostate cancer, testicular cancer, and leukemia.^9^, ^10^, ^11^ Moreover, cancer survivors frequently experience long‐term adverse events related to the cancer treatment such as the development of metabolic syndrome and subsequent cardiovascular disease.^12^, ^13^, ^14^

Evidence is accumulating that physical exercise complementary to cancer treatment is safe and feasible.^15^, ^16^ Encouraging effects of exercise interventions to improve lifestyle in patients with various cancer diagnoses have been reported.^15^, ^17^, ^18^ In general, exercise interventions can alleviate common side effects of cancer treatment, for example, by increasing patients' physical fitness, improving quality of life, and reducing cancer‐related fatigue.^6^, ^7^, ^17^, ^19^ In patients diagnosed with lymphoma, breast cancer, colorectal cancer, or prostate cancer, physical exercise may be associated with improved progression‐free survival.^20^, ^21^, ^22^ Importantly, an increase in physical exercise behavior and maintenance of this behavior after completion of cancer treatment may lower the risk of cancer recurrence, as reported in patients diagnosed with breast or prostate cancer.^23^, ^24^ In various cancer types, physical exercise appears to decrease disease‐related morbidity and mortality.^24^, ^25^, ^26^, ^27^ A meta‐analysis of 23 prospective studies in breast and colorectal cancer survivors found that engaging in at least 150 minutes of moderate to vigorous intensity physical exercise was associated with a reduction in the risk of overall mortality of approximately 24% compared to being less physically active.^24^ These benefits are comparable to the effect of smoking cessation on reducing the risk of cancer mortality.^28^

Behavioral change, focused on adaption of a healthier lifestyle, is complicated. A cancer diagnosis and subsequent treatment may potentially motivate patients to change their lifestyle (eg, to become more active, follow a healthier diet, or quit smoking).^29^, ^30^, ^31^ In observational studies, however, a decrease in patients' physical exercise frequency was found after being diagnosed with breast cancer and this effect was more distinct in obese, sedentary, and elderly patients.^32^, ^33^

Accumulating data on the negative effects of being overweight on the development of cancer and cancer survival fuel the sense of urgency for successful interventions to enhance a healthy lifestyle.^8^, ^21^, ^34^ Unfortunately, low adherence to the interventions and limited recruitment rates are frequently reported in studies investigating exercise interventions in cancer patients, both during and after cancer treatment.^35^, ^36^ Several barriers to physical exercise (eg, fatigue, time restraints, and discomfort) have been reported.^35^, ^37^, ^38^ Understanding which factors predict adherence to exercise interventions is essential to identify patients that are intending to increase their physical exercise intensity but who are at risk of nonadherence. Identifying predictors of exercise adherence can contribute to an increased number of cancer patients participating in exercise interventions, with potential benefits in cancer outcome.^36^, ^39^

The aim of this review is to identify predictors of adherence to exercise interventions in patients with cancer, during and after multimodality cancer treatment. This knowledge will help optimize implementation strategies and eventually help in improving cancer treatment outcome.

## 2 MATERIALS AND METHODS

### 2.1 Design

A systematic review was performed to identify predictors of adherence to exercise interventions from randomized controlled trials (RCTs) and to discuss the methodological quality and results of included papers. This systematic review was conducted in accordance with Preferred Reporting Items of Systematic Reviews and Meta‐Analyses (PRISMA) guidelines.^40^

### 2.2 Literature search

A literature search was performed using electronic databases (PubMed, Embase, and Cochrane) to identify relevant paper published before February 1, 2017. The complete search including Medical Subject Headings (MeSH) terms and keywords is described in Tables S1 and S2. In addition, reference tracking of all papers was performed. Full papers were eligible for inclusion when they reported an RCT design, were conducted in adult cancer patients who participated in a physical exercise intervention during or after systemic (neo‐) adjuvant cancer treatment, provided outcome of factors predicting exercise intervention adherence, and were written in English. An exercise intervention was defined as exercise interventions involving any physical movement produced by skeletal muscles that require energy expenditure^41^; that were planned, structured, and repetitive; that were of at least moderate to vigorous intensity; and that were aimed to improve or maintain physical fitness over a predetermined time period.^42^ Pilot studies, case studies, and papers of low methodological quality were not included.

### 2.3 Selection of studies

Selected papers were screened based on title and abstract. In cases when titles and abstracts implied that a paper was potentially eligible for inclusion, a full paper copy of the report was obtained and evaluated for inclusion.

### 2.4 Data extraction and assessment of methodological quality

Data were extracted using a predetermined extraction form and in accordance with PRISMA guidelines.^40^ Data extracted were as follows: (1) first author's last name, year of publication, country, and trial name; (2) design; (3) population (number of participants, gender, age, cancer type(s), stage, and treatment modalities); (4) exercise intervention (extent, duration, type, frequency, treatment phase, intensity, adherence facilitation, and control group program); (5) outcome (outcome measures of adherence and measurement instruments); (6) results (adherence rate, univariable and multivariable analysis, and variance in exercise intervention adherence explained by analyzed factors (R^2^ or area under the curve [AUC]). Two investigators conducted the search and data extraction in collaboration (G.S. and H.O.). The two investigators scored the methodological quality of included papers independently (G.S. and H.O.) using the Physiotherapy Evidence Database (PEDro) scale.^43^ The scale is composed of 11 items, of which the first item is only applicable for specification of eligibility criteria and is not considered as part of calculating the overall PEDro score. Studies scored one point for each item present and could score between 0 to 10 points. Studies that scored ≥4 points were classified as “high” quality and studies that scored <4 points were considered to be of “low” methodological quality.^44^ Disagreement between the 2 investigators regarding a papers' quality score was resolved by discussion with a third investigator (A.W.) until consensus was reached. Cohen's Kappa and percentage of agreement on methodological quality were calculated.

## 3 RESULTS

### 3.1 Selection of studies

**Figure 1:**
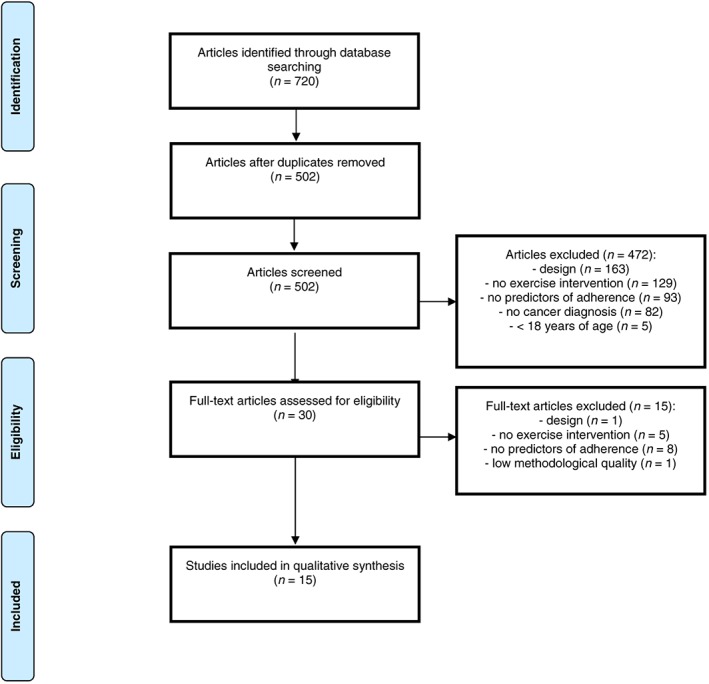
Flowchart of the literature search strategy and study selection in the systematic review

The primary search strategy identified 720 potentially relevant papers, of which 502 remained after discarding duplicates (Figure 1). After screening based on title and abstract, 30 papers were potentially eligible for inclusion. Fifteen of these papers met predefined eligibility criteria, of which the oldest paper was published in 2002.^45^, ^46^, ^47^, ^48^, ^49^, ^50^, ^51^, ^52^, ^53^, ^54^, ^55^, ^56^, ^57^, ^58^, ^59^

### 3.2 Characteristics of included studies

In total, 2279 cancer patients were included in the 15 studies analyzed.^45^, ^46^, ^47^, ^48^, ^49^, ^50^, ^51^, ^52^, ^53^, ^54^, ^55^, ^56^, ^57^, ^58^, ^59^ Of these patients, 1383 were assigned to an exercise intervention and these patients had a mean age of 55.5 years. All studies used an RCT design and were conducted in the United States, Canada, Australia, the Netherlands, Germany, or Taiwan. Eligibility criteria were heterogeneous among studies, with differences in cancer type(s), cancer treatment phase, exercise interventions, and patient characteristics. A full description of the different study characteristics is depicted in Table 1.

**Table 1:** Characteristics of the 15 studies included in the systematic review

Author, Year, Country, Trial Name	Design	Population ([*n*], Gender, Age [±], Cancer Type, Stage, Treatment)	Exercise Intervention (Extent, Type, Frequency, Duration, Intensity), CG Program	Adherence Facilitation	Outcome Measure(s) Adherence, Measurement Instrument(s) Adherence
During treatment, center‐based, or a combined center‐ and home‐based exercise intervention					
Arem, 2016, USA, HOPE study 45	Two‐armed RCT	n = 121 Women 100% 62.0 BC survivors I to III HT > 6 months	‐ Yearlong, supervised and unsupervised multimodal exercise intervention: AET, RET (6 exercises), 2 of the 3 times per week supervised with progressive intensity ‐ Continue usual physical exercise	Education booklet, exercise log, self‐monitoring, supervision by certified cancer exercise trainers	‐ Average minutes of weekly moderate to vigorous AET; percentage attended prescribed supervised RET ‐ Objective attendance; exercise log
Courneya, 2014, Canada, CARE trial^46^	Three‐armed RCT	n = 301 Women 100% 50.0 BC Stage I to IIIa Initiating adjuvant CT, S, CT, COT	‐ Duration of CT, randomization in a supervised AET or AET and RET (9 exercises) group, 3 times a week with progressive AET intensity and duration ‐ Continue usual physical exercise	Tailoring of exercise prescription to patients' response to exercise and CT	‐ Percentage attended prescribed supervised exercise sessions ‐ Objective attendance
Courneya, 2008, Canada, START trial^47^	Three‐armed RCT	n = 242 Women 100% 49.2 BC Stage I to IIIA Adjuvant CT	‐ Duration of CT, supervised multimodal exercise intervention: AET, RET (9 exercises)*,* 3 times a week with progressive intensity and duration ‐ Asked not to initiate an exercise intervention	Availability of open training facilities, tailoring of exercise prescription	‐ Percentage attended prescribed supervised exercise sessions ‐ Objective attendance
Courneya, 2004a, Canada, no trial name^48^	Two‐armed RCT	n = 155 Women 0% 68.0 PC Stage I to IV ADT	‐ 12‐wk supervised RET (9 exercises), 3 times a week with progressive intensity and duration ‐ Continue usual physical exercise	Supervision by experienced fitness consultants, positive feedback, tailoring of exercise prescription	‐ Number of attended prescribed exercise sessions ‐ Objective attendance
During treatment, home‐based exercise intervention					
Shang, 2012, USA, no trial name^49^	Two‐ armed RCT	n = 126 Women 38.9% 60.2 BC, CRC, PC, others Stage 0 to III RT, CT, COT, BT	‐ 5‐ to 35‐wk home‐based AET, 2 to 5 times a week with progressive intensity and duration *‐* Continue usual physical exercise	Telephone calls biweekly, adjusting walking prescription according to patients' condition, self‐monitoring	‐ Percentage adherent weeks of prescribed physical exercise (> 60 min physical exercise in 3 sessions) ‐ Pedometers, exercise log
After treatment, center‐based, or a combined center‐ and home‐based exercise intervention					
McNeely, 2012, Canada, no trial name^50^	Two‐armed RCT	n = 52 Women 29% 52 HNC Stage 0 to IV S, RT, CT	‐ 12‐wk supervised active and passive range of motion/stretching exercises, postural exercises, and strengthening exercises with progressive intensity and duration ‐ Standard exercise intervention, low‐intensity resistance exercise training	Tailoring of exercise prescription	‐ Percentage attended prescribed supervised exercise sessions‐ Objective attendance
McGuire, 2011, USA, no trial name^51^	Two‐armed RCT	n = 223 Women 100% 58.7 Postmenopausal BC survivors Stage 0 to II S, RT, CT	‐ 24*‐*mo resistance training 0 to 8 months home‐based, 9 to 24 center‐based, 2 times a week (9 exercises) with progressive intensity and duration ‐ Medication only, standard care	Education, goal‐setting, feedback, encouragements, tailoring of exercise	‐ Percentage prescribed number of exercises performed ‐ Exercise log
After treatment, home‐based exercise intervention					
Kampshoff, 2016, The Netherlands, REACT study^52^	Three‐armed RCT	n = 277 Women 80% 53.5 BC, CRC, PC, OC, CC, TC, LY Local/advanced RT, CT, IT, COT, HT, S	‐ 4‐6 wk after completion of primary cancer treatment, 12 wk of supervised AET and RET (6 exercises), randomization in a high intensity or low to moderate intensity group, 2 times a week with progressive AET and RET intensity and duration *‐* Waiting list CG	Personalized feedback, tailoring of exercise, addressing patients' barriers to exercise	‐ Percentage attended prescribed supervised exercise sessions; average minutes of weekly moderate‐ to vigorous AET; percentage of prescribed RET training load ‐ Exercise log and objective attendance
Latka, 2009, USA, Yale Exercise and Survivorship Study^53^	Two‐armed RCT	n = 75 Women 100% 55.8 BC survivors Stage 0 to IIIA RT, CT, COT, HT, S	‐ 6‐mo home‐based moderate‐intensity AET, 3 times a week at health club and 2 times a week at home with progressive intensity and duration ‐ Continue usual physical exercise	Personalized feedback, tailoring of exercise, self‐monitoring, goal setting, weekly counseling, quarterly newsletters	‐ Average minutes of weekly moderate‐ to vigorous physical exercise ‐ Exercise log
Pinto, 2009, USA, MF trial^54^	Two‐armed RCT	n = 86 Women 100% 53.4 BC survivors Stage 0 to II RT, CT, HT, S	‐ 12‐wk home‐based moderate intensity AET, AET starting at 2 to 5 times a week at the end with progressive intensity and duration *‐* Continue usual physical exercise	Self‐monitoring, weekly telephone calls, sending encouraging letters, addressing patients' barriers to exercise, tailoring of exercise	‐ Average minutes of weekly moderate‐ to vigorous physical exercise, meeting weekly exercise goals ‐ Exercise log, pedometer, weekly exercise goals
During and after treatment, center‐based, or a combined center‐ and home‐based exercise intervention					
Kuehl, 2016, Germany, PETRA study^55^	Two‐armed RCT	n = 147 Women 32.7% 53.8 AML, ALL, LY/ CLL, MDS, CML/MPS, MM, other Stage I to III CT, RT, allo‐HCT	‐ 24*‐*mo partly supervised and unsupervised multimodal exercise intervention during treatment: AET, RET, 3 to 5 times per week with progressive intensity RET and AET after discharge patients continued the exercise intervention at home ‐ Muscle relaxation intervention	Tailoring of exercise*,* (bi‐)weekly advice for home‐based exercise, encouragements	‐ Average minutes of weekly moderate‐ to vigorous physical exercise ‐ Exercise log
Craike, 2016, Australia, ENGAGE study^56^	Two‐armed RCT	n = 147 Women 0% 66.9 PC Stage I to III S, ADT, RT	‐ 12‐wk supervised and unsupervised multimodal exercise intervention: AET, RET (6 exercises), balance and flexibility exercises, 2 of the 3 times per week supervised with progressive intensity ‐ Usual care physical exercise advice	Tailoring of exercise*,* weekly advice for home‐based exercise, discussions, goal setting, addressing patients' barriers to exercise, strategies	‐ Percentage attended prescribed supervised exercise sessions ‐ Objective attendance
Courneya, 2010, Canada, HELP trial^57^	Two‐armed RCT	n = 122 Women 41.0%) 53.2 LY Stage 0 to IV RT, CT	‐ 12‐wk supervised AET, 3 times a week with progressive intensity and duration ‐ Asked not to exceed baseline physical exercise	Planned exercise sessions, telephone follow‐up after missed sessions, encouragements, paid parking, tailoring of exercise	‐ Percentage attended prescribed supervised exercise sessions‐ Objective attendance
During and after treatment, home‐based exercise intervention					
Courneya, 2004b, Canada, CAN‐HOPE trial^58^	Two‐armed RCT	n = 93 Women 41.9% 60.3 CRC Stage I to IV S, RT, CT, COT	‐ 4‐mo home‐based AET (eg, walking, swimming), 3 to 5 times a week with progressive intensity and duration ‐ Asked not to initiate an exercise intervention	Telephone calls, addressing patients' exercise barriers, encouragements, tailoring of PA	‐ Average minutes of weekly moderate‐ to vigorous physical exercise ‐ LSI
Courneya, 2002, Canada, GROUP‐HOPE trial^59^	Two‐armed RCT	n = 96 Women 84.4% 51.6 BC, CRC, LY, others Stage I to IV S, RT, CT, COT	‐ 4‐mo home‐based AET (eg, walking, swimming), 3 to 5 times a week with progressive intensity and duration *‐* Continue usual physical exercise	Telephone calls, answering questions, tailoring of exercise	‐ Average minutes of weekly moderate‐ to vigorous physical exercise ‐ LSI

Abbreviations: CG, control group; RCT, randomized controlled trial; BC, breast cancer; CRC, colorectal cancer; PC, prostate cancer; LY, lymphoma; HNC, head and neck cancer; OC, ovarian cancer; CC, cervix cancer; TC, testis cancer; AML, acute myeloid leukemia; ALL, acute lymphoblastic leukemia; CLL, chronic lymphocytic leukemia; MDS, myelodysplastic syndrome; CML, chronic myeloid leukemia; MPS, myeloproliferatory syndrome; MM, Multiple myeloma; CT, chemotherapy; RT, radiation therapy; BT, brachy therapy; COT, combined therapy; HT, hormone therapy; S, surgery; IT, immune therapy; ADT, androgen deprivation therapy; allo‐HCT, allogeneic stem cell transplantation; AET, aerobic exercise training; RET, resistance exercise training; LSI, leisure time index.

Applied timing of exercise interventions varied. Five studies applied their exercise intervention after systemic (neo‐) adjuvant treatment,^50^, ^51^, ^52^, ^53^, ^54^ 5 studies both during and after treatment,^55^, ^56^, ^57^, ^58^, ^59^ and 5 studies during treatment.^45^, ^46^, ^47^, ^48^, ^49^ Furthermore, 4 studies included a population with multiple cancer types,^49^, ^52^, ^55^, ^59^ whereas 11 other studies included a single cancer type population.^45^, ^46^, ^47^, ^48^, ^50^, ^51^, ^53^, ^54^, ^56^, ^57^, ^58^ Six of the 15 studies included only patients diagnosed with breast cancer,^45^, ^46^, ^47^, ^51^, ^53^, ^54^ 2 studies included patients with prostate cancer,^48^, ^56^ 1 study included patients with head and neck cancer,^50^ 1 study included patients with lymphoma,^57^ and 1 study included patients with colorectal cancer.^58^

In 5 studies, the exercise intervention was performed at a rehabilitation center (center based)^46^, ^47^, ^48^, ^50^, ^57^; in 6 studies, the intervention was performed at the patient's home (home based)^49^, ^52^, ^53^, ^54^, ^58^, ^59^; and 4 studies conducted their intervention in both settings.^45^, ^51^, ^55^, ^56^ Duration of exercise interventions ranged from 5 weeks to 24 months. Various physical exercise modalities were used in the selected studies: aerobic (brisk walking, cycling, treadmill, or swimming),^53^, ^54^, ^57^, ^58^, ^59^ strength (resistance, stretching, and postural exercises),^48^, ^50^, ^51^ or combined aerobic and strength exercises.^45^, ^46^, ^47^, ^49^, ^52^, ^55^, ^56^

Intensity of the exercise interventions differed from low to high (high intensity in terms of exercise sessions that were more frequent, of longer duration or with a higher peak oxygen uptake [VO_2_ peak percentage]). All studies conducted the exercise interventions with progressive intensity, and in nearly all studies, physiotherapists or exercise physiologists tailored the exercise interventions to the patient's health by modifying exercise prescriptions. Additionally, patients' adherence to exercise was facilitated in all studies.

### 3.3 Assessment of methodological quality and quantitative analysis

The 15 included studies were scored using the PEDro scale. The 2 investigators (G.S. and H.O.) agreed on 147 of the maximal 160 points (91.9%). Kappa statistics calculated for agreement of the methodological quality assessment between the 2 investigators was 0.82, corresponding with an excellent agreement. Methodological quality ranged from 4 to 8 as rated on the PEDro scale with a median score of 7 of 10, confirming “high” methodological quality. All studies were rated as high qualitative studies^45^, ^46^, ^47^, ^48^, ^49^, ^50^, ^52^, ^53^, ^54^, ^55^, ^56^, ^57^, ^58^, ^59^ with a score of ≥4, of which 6 scored 8 of 10 points.^48^, ^50^, ^52^, ^53^, ^58^, ^59^ The methodological quality assessment is summarized in Table 2. Unfortunately, a quantitative analysis by pooling outcome data (meta‐analysis) or a best‐evidence synthesis was inappropriate. This is due to incomparability of outcome data caused by heterogeneity of study sample characteristics (eg, divergent exercise interventions, patient characteristics, and outcome as summarized in Table 1).

**Table 2:** Methodological quality of the 15 studies included in the systematic review

Author, Year	Randomization	Concealed Allocation	Group Similarity at Baseline	Blinding of Patients	Blinding of Therapists	Blinding of Assessors	Obtained Measures of >85% of ≥1 Outcome	Intention‐to‐treat Analysis	Between‐ group Statistical Comparisons	Point Measure; Variability of Data	Total
Arem, 2016^45^	+	‐	+	‐	‐	‐	+	+	+	+	6/10
Courneya, 2014^46^	+	+	+	‐	‐	‐	+	+	+	+	7/10
Courneya, 2008^47^	+	+	+	‐	‐	‐	+	+	+	+	7/10
Courneya, 2004a^48^	+	+	+	‐	‐	+	+	+	+	+	8/10
Shang, 2012^49^	+	‐	+	‐	‐	‐	+	+	+	+	6/10
McNeely, 2012^50^	+	+	+	‐	‐	+	+	+	+	+	8/10
McGuire, 2011^51^	+	‐	+	‐	‐	‐	‐	+	+	+	5/10
Kampshoff, 2016^52^	+	+	+	‐	‐	+	+	+	+	+	8/10
Latka, 2009^53^	+	+	+	‐	‐	+	+	+	+	+	8/10
Pinto, 2009^54^	+	‐	+	‐	‐	‐	+	+	+	+	6/10
Kuehl, 2016^55^	+	+	‐	‐	‐	‐	‐	+	‐	+	4/10
Craike, 2016^56^	+	+	+	‐	‐	‐	+	+	+	+	7/10
Courneya, 2010^57^	+	+	+	‐	‐	‐	+	+	+	+	7/10
Courneya, 2004b^58^	+	+	+	‐	‐	+	+	+	+	+	8/10
Courneya, 2002^59^	+	+	+	‐	‐	+	+	+	+	+	8/10
Total	15/15	11/15	14/15	0/15	0/15	7/15	13/15	15/15	14/15	15/15	

Abbreviations: +, positive quality assessment; ‐, negative quality assessment.

### 3.4 Measurement instruments and outcome measures of adherence

In 7 studies, adherence to exercise intervention was measured using an exercise log.^45^, ^49^, ^51^, ^52^, ^53^, ^54^, ^55^ In 2 studies, patients used a pedometer to measure adherence.^49^, ^54^ Seven studies assessed adherence through recording of attended exercise intervention sessions.^45^, ^46^, ^47^, ^48^, ^50^, ^56^, ^57^ An alternative instrument to record adherence, applied by 2 studies, was the leisure score index.^58^, ^59^ One study assessed adherence by verifying whether patients met their weekly exercise goals^54^ (Table 1).

Outcome of adherence to exercise intervention was defined by 7 studies as percentage of scheduled minutes of weekly moderate to vigorous physical exercise.^45^, ^52^, ^53^, ^54^, ^55^, ^58^, ^59^ Ten studies defined exercise intervention adherence as number or percentage of attended exercise sessions.^45^, ^46^, ^47^, ^48^, ^49^, ^50^, ^51^, ^52^, ^56^, ^57^ Two studies defined adherence as a number of steps per week.^49^, ^54^ One study defined adherence as meeting the weekly exercise goal(s).^54^ One study defined adherence as percentage of prescribed intensity, frequency, and duration of the multimodal (resistance and aerobic) exercise intervention^52^ (Table 1).

### 3.5 Univariable and multivariable analyses of selected studies

A wide range of predictive factors were investigated, which were classified as socio‐demographic (eg, gender, marital status, education, employment, location of the rehabilitation center in relation to the residential area, family support, and feedback by trainers), medical (eg, cancer type, treatment regimen, pretreatment fatigue, and disease stage), physical and physiological (eg, physical fitness and body mass index) and behavioral factors (eg, exercise history, baseline self‐efficacy, exercise motivation, smoking behavior, and alcohol consumption). Study results are depicted in Tables 3 and S3.

**Table 3:** Overview of significant predictors of exercise intervention adherence found in multivariable analysis

	During Treatment		After Treatment	
Exercise Intervention Adherence	High	Low	High	Low
Socio‐demographic factors				
Being married		49		51
Gender (male)	59, a		59, a	
Close location/center	46, 47			
Having children at home	55, a			
More knowledge and skills of exercises			51	
High intensity exercise group assignment		46		
More family support			51	
More feedback by trainers			51	
Low employment status		58 a		58 a
Medical factors				
Extensive treatment protocol		46 ^;^ 58 a		50 ^;^ 58 a
Pretreatment fatigue		49 ^;^ 55, a		
Advanced disease stage		47		
Cancer types other than breast cancer				52
Low psychological distress			52	
Exercise limitations due to cancer treatment		46		
Endocrine symptoms		46		
High depression		46, 47		
Physiological and physical factors				
High physical fitness	49			
High age	45 ^;^ 57 a	48	57 a	
High VO_2_ peak	45, 46, 47			
High submaximal endurance capacity			55, a	
Low BMI			53	
Behavioral factors				
High exercise stage of change	48			
High exercise history	57 a		54 ^;^ 55, a ^;^ 57 a ^;^ 59, a	
High self‐efficacy			52 ^;^ 54	
Being a nonsmoker			52	
High previous exercise adherence			51 ^;^ 55, a	
High alcohol consumption				50
High exercise motivation	58 a		53 ^;^ 58 a	
High role functioning	56 a		56 a	
High mid‐treatment mood disturbance		49		

a Exercise intervention covered both time periods, during and after treatment.

Cancer type: Black, multiple cancer types; Red, breast cancer; Blue, prostate cancer; Purple, head and neck cancer; Orange, lymphoma; Green, colorectal cancer.

Abbreviations: VO_2_ peak, peak rate of oxygen consumption during incremental exercise; BMI, body mass index.

Highly significant (*P* ≤ .01) and significant (*P* < .05) or borderline significant (*P* < .10) associations between exercise intervention adherence and various factors were identified in univariable^45^, ^46^, ^47^, ^48^, ^50^, ^52^, ^53^, ^55^, ^56^, ^57^, ^58^, ^59^ or bivariable analysis.^49^, ^51^ Thereafter, these factors were included in a multivariable analysis to finally derive predictors of adherence to exercise intervention. One study did not describe a univariable or bivariable analysis.^54^ An overview of the significant predictors of adherence to exercise interventions during and after cancer treatment is summarized in Table 3. Adherence rates ranged from 61.9% to 91.0%. The R^2^, defined as the percentage of variance explained by the model, was reported in 9 studies^46^, ^47^, ^48^, ^49^, ^50^, ^51^, ^55^, ^57^, ^58^, ^59^ and ranged from 20.4% to 75.0%. One study described the fit of the multivariable model by calculating the area under the curve, with reported values^52^ of 0.67 to 0.75. Data and *P* values of univariable and multivariable analyses are summarized in Table S3. All factors gathered in multivariable analysis in each study were summarized and weighted as a predictor of exercise adherence. Factors found in the multivariable analyses that significantly predicted adherence to exercise intervention in cancer patients during and after cancer treatment are presented in Table 3.

## 4 DISCUSSION

This review summarizes predictors of adherence to exercise intervention by patients during and after multimodality cancer treatment. Adherence to exercise interventions varies among trials.^50^, ^51^, ^52^, ^53^ Insight in factors determining adherence can optimize exercise intervention implementation strategies and eventually improve cancer treatment outcome. The most important result is that adherence to exercise depends on different factors during different stages of cancer treatment and in different cancer types. More specifically, medical factors predicting low adherence to exercise interventions during treatment include advanced disease stage, extensive treatment protocols, and exercise limitations due to cancer treatment (Table 3). Factors predicting high adherence to the exercise intervention after treatment include socio‐demographic, physical, physiological, and behavioral factors; more family support and feedback by trainers, physical fitness, high self‐efficacy, high motivation to exercise and being a non‐smoker. To enhance adherence to exercise interventions during and after treatment, it would be most beneficial to address behavioral factors and socio‐demographic factors.^60^ Examples include providing exercise interventions close to the patient's home, stimulating family support and increasing exercise motivation by improving feedback and coaching by trainers.

The location of the rehabilitation center contributes highly in predicting adherence to center‐based exercise interventions during chemotherapy in breast cancer patients, as described by 2 studies of Courneya et al.^46^, ^47^ Reduced travel distance between the residential area and rehabilitation center was previously identified as a predictor of better adherence to exercise intervention in pediatric patients diagnosed with cancer.^61^ Likewise, prolonged travel distance was found to be a predictor of worse adherence to a supervised exercise intervention in patients with chronic obstructive pulmonary disease who were rehabilitated in an 8‐week supervised exercise intervention.^62^ Participation in exercise interventions is time‐consuming, especially when patients rely on public transportation for travelling to the exercise location.^63^ Travel distance not only negatively influences exercise adherence, it is often a reason to not participate in center‐based exercise interventions.^47^, ^61^ Albornoz et al highly recommend distribution of treatment locations throughout the country and thus near patients' homes.^64^

Home‐based exercise interventions, in which patients can exercise individually, could offer a convenient solution and may be preferred by certain groups of patients, eg, when travel distances are long.^65^, ^66^ However, a disadvantage of these home‐based interventions is that control of exercise adherence is suboptimal. Supervision or coaching in the home‐based setting is based on enhancing exercise adherence by stimulating family support and feedback by physiotherapists and improving exercise knowledge and skills of exercise.^46^, ^67^ In addition, upcoming technological developments, eg, tools such as wearable activity trackers and mobile applications, facilitate objective monitoring of patients' exercise adherence in home‐based settings.^68^ These tools can measure and record exercise levels, which can help monitor patients' physical exercise behavior after completing a supervised exercise intervention.^25^

Higher willingness to change physical exercise behavior, ie, exercise motivation, was a significant predictor in 4 of the included studies.^48^, ^53^, ^58^, ^59^ This is in line with the meta‐analysis performed by Husebø et al, in which a significant association between exercise motivation and exercise intervention adherence was described.^69^ Exercise motivation is measured by the transtheoretical model stage of behavior change, one of many behavioral models used in exercise motivation.^70^ This model describes motivational processes involved in attempting to change physical exercise behavior, including the stages of precontemplation, contemplation, preparation, action, and maintenance. According to the meta‐analysis performed by Marshall et al, transition from the precontemplation phase (sedentary, no intention) to the contemplation phase (sedentary, intention within 6 mo) may especially contribute to a change in behavior.^71^ This result suggests that facilitating behavioral change after cancer diagnosis could result in improved exercise adherence. However, behavioral factors are more crucial in predicting exercise adherence in unsupervised exercise compared to supervised interventions.^47^

Awareness of the importance of physical exercise not only in cancer treatment but also in other chronic diseases, such as chronic obstructive pulmonary disease and diabetes, has increased over the past years.^72^, ^73^ Consequently, the number of RCTs investigating exercise interventions during and after cancer treatment has increased. However, data on predictors of adherence to the exercise intervention are often not described in these RCTs, particularly in those performed in a home‐based setting.^74^

One of the strengths of this systematic review is that all studies were of “high” methodological quality. This is in contrast to methodological quality assessment of a previous systematic review, assessing determinants of exercise adherence and maintenance.^75^ This difference may be due to the use of a methodological quality assessment tool that was adapted from existing quality criteria lists compiled by Kampshoff et al,^75^ whereas we pursued the PRISMA guidelines for reporting systematic reviews and used the PEDro scale, which is especially designed for assessment of clinical trials.^40^, ^43^ The internal validity of our review is partially warranted by limiting the inclusion to randomized studies.^76^

A systematic review by Husebø et al demonstrated that several psychological factors predicted exercise intervention adherence. However, socio‐demographic, medical or physical, and physiological factors were not investigated.^69^ In contrast, our review indicated that psychological factors only partially predicted exercise intervention adherence and suggest a more important role for socio‐demographic, medical or physical, and physiological factors, such as fewer exercise limitations due to cancer treatment, pretreatment fatigue or high VO_2_ peak levels.

### 4.1 Study limitations

A limitation of our review was the relatively low number of RCTs included despite the extensive literature search. Few RCTs that investigated predictors of exercise intervention adherence during and after cancer treatment and met our inclusion criteria were identified. Grey literature was not considered in the literature search. The possibility that an RCT fulfilling our inclusion and exclusion criteria was conducted but not reported in the scientific literature was estimated to be very small. We were unable to perform a quantitative analysis or a best‐evidence synthesis, due to the heterogeneity of the data.

### 4.2 Clinical implications and conclusions

In summary, recommendations for future trials include the use of equivalent measuring instruments in future RCTs to facilitate a more homogeneous analysis across studies. We recommend future RCTs to report predictors of exercise intervention adherence and to use objective measurement instruments such as attendance records and validated wearable activity trackers (eg, accelerometers). This facilitates the comparison of studies investigating predictors of exercise intervention adherence during and after multimodality cancer treatment.^74^ Hence, the power of generated data in the field of exercise oncology will increase. Furthermore, we recommend the analysis and reporting of potential preexistent factors that may impede adherence to and participation in an exercise intervention in clinical practice. In this manner, patients less likely to adhere can be offered a personalized exercise intervention and extra guidance, by means of, eg, prolonged coaching to facilitate exercise adherence.^77^, ^78^ These approaches might result in optimizing participation in exercise interventions and retaining the less motivated, less fit patients who will potentially benefit most.^25^ Since it is increasingly recognized that exercise interventions should be included in the treatment of cancer patients, predictors of exercise intervention adherence should be taken into account when composing these interventions.

## CONFLICT OF INTEREST

The authors have no funding or conflicts of interest to disclose.

## ETHICS APPROVAL

As a systematic review, no ethical approval was sought for this article.

## Supporting information

Table S1. Search string

Table S2. Keywords and phrases

Table S3. Main results of the 15 studies included in the systematic review
