# Supplementary material for: Predictors of adherence to exercise interventions during and after cancer treatment: A systematic review
Source: Psychooncology. 2018 Jan 26;27(3):713–24. doi: 10.1002/pon.4612 (PMC5887924; doi:10.1002/pon.4612)
Supplement: Supplementary file 1 — Table S1. Search string [file PON-27-713-s001.doc]

Supplementary Table 1. Search string

| Database | Search string | Hits |
| --- | --- | --- |
| Pubmed | (((((((("Cancers"[Title/Abstract]) OR “Cancer”[Title/Abstract]) OR “neoplasms”[Title/Abstract]) OR “neoplasm”[Title/Abstract]) OR “neoplasms”[MeSH Terms])) AND ((((((((((("Motor activity"[MeSH Terms]) OR "Strength training"[Title/Abstract]) OR "Exercise"[MeSH Terms]) OR "Exercise training"[Title/Abstract]) OR "Exercise therapy"[MeSH Terms]) OR "Exercise therapy"[Title/Abstract]) OR "Exercise program"[Title/Abstract]) OR "Physical exercise"[Title/Abstract]) OR "Occupational therapy"[MeSH Terms]) OR "Exercise intervention"[Title/Abstract]) OR "Physical activity"[Title/Abstract])) AND ((((((((((("Patient compliance"[MeSH Terms]) OR "Patient dropouts"[MeSH Terms]) OR "Dropouts"[Title/Abstract]) OR “Attendance”[Title/Abstract]) OR "Dropout"[Title/Abstract]) OR "Adherence"[Title/Abstract]) OR "Compliance"[Title/Abstract]))))) AND (((((((((("Prognosis"[MeSH Terms]) OR "Prognosis"[Title/Abstract]) OR "Predictor"[Title/Abstract]) OR “Determinants”[Title/Abstract]) OR “Correlates”[Title/Abstract]) OR "Predictors"[Title/Abstract]) OR "Predictive value of tests"[MeSH Terms]) OR "Predictive"[Title/Abstract])))) | 249 |
| Cochrane Library | ([mh “neoplasms”] OR "Cancer":ti,ab OR "Cancers":ti,ab OR "Neoplasms":ti,ab OR "Neoplasm":ti,ab) AND ([mh “Motor activity”] OR "Strength training":ti,ab OR "Exercise training":ti,ab OR [mh “Rehabilitation”] OR "Exercise therapy":ti,ab OR "Exercise program":ti,ab OR "Physical exercise":ti,ab OR "Exercise intervention":ti,ab OR "Physical activity":ti,ab) AND (“Attendance”:ti,ab OR [mh “Patient compliance”] OR [mh “Patient dropouts”] OR "Dropouts":ti,ab OR "Dropout":ti,ab OR "Adherence":ti,ab OR "Compliance":ti,ab) AND ([mh “Prognosis”] OR "Prognosis":ti,ab OR "Predictors":ti,ab OR "Predictor":ti,ab OR "Correlates":ti,ab OR "Determinants":ti,ab OR [mh “Prospective studies”] OR [mh “Predictive value of tests”] OR "Predictive":ti,ab) | 156 |
| EMBASE | ("Neoplasm"/exp OR "neoplasms":ab,ti OR "neoplasm":ab,ti OR "Cancer":ab,ti OR "Cancers":ab,ti) AND ("Motor activity"/exp OR "Strength training":ab,ti OR "Exercise"/exp OR "Exercise training":ab,ti OR "Kinesiotherapy"/exp OR "Exercise therapy":ab,ti OR "Exercise program":ab,ti OR "Physical exercise":ab,ti OR "Occupational therapy"/exp OR "Exercise intervention":ab,ti OR "Physical activity":ab,ti) AND ("Patient compliance"/exp OR "Patient dropouts"/exp OR "Attendance":ab,ti OR "Dropouts":ab,ti OR "Dropout":ab,ti OR "Adherence":ab,ti OR "Compliance":ab,ti) AND ("Prognosis"/exp OR "Prognosis":ab,ti OR "Predictors":ab,ti OR "Predictor":ab,ti OR "Correlates":ab,ti OR "Determinants":ab,ti OR "Forecasting"/exp OR "Prediction"/exp OR "Predictive value"/exp OR "Predictive":ab,ti) | 305 |
|  | Total number of citations retrieved | 720 |
|  | Number of duplicates | 218 |
|  | Total number of citations, duplicates discarded | 502 |
|  | Number discarded after screening title, abstract | 472 |
|  | Total number of citations after screening title, abstract | 30 |
|  | Number discarded after reading full text | 15 |
|  | Total number of citations after reading full text | 15 |
