# Supplementary material for: Predictors of adherence to exercise interventions during and after cancer treatment: A systematic review
Source: Psychooncology. 2018 Jan 26;27(3):713–24. doi: 10.1002/pon.4612 (PMC5887924; doi:10.1002/pon.4612)
Supplement: Supplementary file 2 — Table S2. Keywords and phrases [file PON-27-713-s002.doc]

Supplementary Table 2. Keywords and phrases

| Domain | Determinant | Outcome (1) | Demarcation (2) |
| --- | --- | --- | --- |
| “Neoplasms” (MeSH) | “Motor activity” (MeSH) | “Patient compliance” (MeSH) | “Prognosis” (MeSH) |
| “Cancer” (Title, abstract) | “Exercise therapy” (MeSH) | “Patient dropouts” (MeSH) | “Prospective studies” (MeSH) |
| “Cancers” (Title, abstract) | “Occupational therapy” (MeSH) | “Exercise intensity” (Title, abstract) | “Predictive value of tests” (MeSH) |
| “Neoplasms” (Title, abstract) | “Exercise” (MeSH) | “Attendance” (Title, abstract) | “Predictive” (Title, abstract) |
| “Neoplasm” (Title, abstract) | “Exercise program” (Title, abstract) | “Adherence” (Title, abstract) | “Prognosis” (Title, abstract) |
|  | “Physical exercise” (Title, abstract) | “Dropouts” (Title, abstract) | “Predictors” (Title, abstract) |
|  | “Exercise intervention” (Title, abstract) | “Compliance” (Title, abstract) | “Predictor” (Title, abstract) |
|  | “Exercise therapy” (Title, abstract) | “Dropout” (Title, abstract) | “Determinants” (Title, abstract) |
|  | “Exercise training” (Title, abstract) |  | “Correlates” (Title, abstract) |
|  | “Physical activity” (Title, abstract) |  |  |
|  | “Strength training” (Title, abstract) |  |  |
