# Supplementary material for: Predictors of adherence to exercise interventions during and after cancer treatment: A systematic review
Source: Psychooncology. 2018 Jan 26;27(3):713–24. doi: 10.1002/pon.4612 (PMC5887924; doi:10.1002/pon.4612)
Supplement: Supplementary file 3 — Table S3. Main results of the 15 studies included in the systematic review [file PON-27-713-s003.doc]

Supplementary Table 3. Main results of the 15 studies included in the systematic review

| Author, year, country, trial name | Design | | Population ((*n*), gender, age (*±)*, cancer type, stage, treatment) | Results 1 (adherence rate, univariable analysis (*P*)) | | | Results 2 (multivariable analysis (*P*), R^2^) | |  |
| --- | --- | --- | --- | --- | --- | --- | --- | --- | --- |
| **During treatment*,*** *center-based or a combined center- and home-based exercise intervention* | | | | | | | | |  |
| Arem, 2016, USA, HOPE study [45] | Two-armed RCT | | *Population* (*n*) T: *n*=121, I: *n*=61, C: *n*=60 *Gender (%)* Women 100% *Age* T: 61, I: 62.0, C: 60.5 *Cancer type* BC survivors *Stage* 0 to III *Treatment* HT>6 months | *Adherence rate* 70% *Univariable analysis at 6 months - AET (mean min/wk) adherence:* ↑Education (*P*=0.022)*; ↑VO_2_peak (*P*=0.004)**; ↓BMI (*P*<0.001)**; ↑years on HT (*P*=0.079) *- RET (% attendance) adherence:* ↑Age (*P*=0.066); ↑education (*P*=0.069); ↑radiation (*P*=0.038)*; ↓BMI (*P*=0.060); ↑baseline physical exercise (*P*=0.004)**; ↑years on HT (*P*<0.001)** *At 12 months - AET (mean min/wk) adherence:* ↑Age (*P*=0.081); ↑race (white) (*P*=0.099); ↑education (*P*=0.003)**; ↑VO_2_peak (*P*=0.017)*; ↓BMI (*P*<0.001)** *- RET (% attendance) adherence:* ↑Age (*P*=0.017)*; ↑education (*P*=0.017)*; ↑unmarried (*P*=0.085); ↓BMI (*P*=0.005)**; ↑baseline physical exercise (*P*=0.016)*; ↑years on HT (*P*=0.009)** | | | *Multivariable analysis (GLM) at 6 months* *- AET (mean min/wk) adherence:* ↑VO_2_peak (*P*=0.033)* *At 12 months - AET (mean min/wk) adherence:* ↑Education (*P*=0.094), ↓BMI (*P*=0.087) *- RET (% attendance) adherence:* ↑Age (*P*=0.025)*, ↑education (*P*=0.089) *R^2^* N/a | |  |
| Courneya, 2014, Canada, CARE trial [46] | Three-armed RCT | | *Population* (*n*) T: *n*=301, STAN: *n*=96, HIGH: *n*=101, COMB *n*=104 *Gender (%)* Women 100% *Age* T: 50.0, STAN: 49.2, HIGH: 50.1, COMB: 50.5 *Cancer type* BC *Stage* I to IIIa *Treatment* Initiating adjuvant CT, S, CT, COT | *Adherence rate* 73% *Univariable analysis (PCC)* ↑Location/center (r=0.42; *P<*0.001)**, ↓group assignment (r=-0.13; *P=*0.025)*, ↑baseline aerobic exercise (r=0.14; *P=*0.015)*, ↓exercise limitations due to cancer treatment (r=-0.10; *P=*0.098), ↓length of CT protocol (r=-0.13; *P=*0.031)*, ↓FEC-D (r=-0.14; *P=*0.013)*, ↓endocrine symptoms (r=-0.17; *P=*0.003)**, ↑VO_2_peak (r=0.16; *P=*0.006)**, ↑peak rating of perceived exertion (r=0.27; *P<*0.001)**, ↑peak respiratory exchange ratio (r=0.31; *P<*0.001)**, ↑leg strength (r=0.25; *P<*0.001)**, ↓body fat,% (r=-0.20; *P<*0.001)**, ↑lean body mass (r=0.16; *P<*0.001)**, ↑physical well-being (r=0.12; *P=*0.036)*, ↑neuropathy symptoms (r=0.17; *P=*0.03)* | | | *Multivariable analysis* ↑Location/center (β=0.41; *P<*0.001)**, ↓endocrine symptoms (β=0.14; *P=*0.009)**, ↓group assignment (β=-0.13; *P=*0.009)**, ↓exercise limitations due to cancer treatment (β=-0.13; *P=*0.009)**, ↓length of CT protocol (β=-0.12; *P=*0.015)*, ↑VO_2_peak (β=-0.12; *P=*0.017)* *R^2^* 26.4% | |  |
| Courneya, 2008, Canada, START trial [47] | Three-armed RCT | | *Population* (*n*) T: *n*=242, C: *n*=82, RET: *n*=82, AET: *n*=78 *Gender (%)* Women 100% *Age* T: 49.2, C: 49.0, RET: 49.5, AET: 49.0 *Cancer type* BC *Stage* I to IIIA *Treatment* Adjuvant CT | *Adherence rate* 70.2% *Univariable analysis (PCC)* ↑Location/center (r=0.30; *P<*0.001)**, ↑VO_2_peak (r=0.21; *P=*0.008)**, ↑muscular strength (r=0.21; *P=*0.008)**, ↓body fat (r=-0.21; *P=*0.012)*, ↓advanced disease stage (r=0.17; *P=*0.031)*, ↑education (r=0.15; *P=*0.053), ↓depression (r=-0.14; *P=*0.073), ↓smoking (r=-0.14; *P=*0.081) | | | *Multivariable analysis* ↑Location/center (β=0.28; *P=*0.001)**, ↑VO_2_peak (β=0.19; *P=*0.016)*, ↓advanced disease stage (β=0.18; *P=*0.015)*, ↓depression (β=-0.16; *P=*0.033)*, ↑muscular strength (β=0.04; *P=*0.666), ↑education (β=0.07; *P=*0.360), ↓smoking (β=-0.08; *P=*0.282) *R^2^*21% | |  |
| Courneya, 2004a, Canada, no trial name [48] | Two-armed RCT | | *Population* (*n*) T: *n*= 155, I: *n*=82, C: *n*=73 *Gender (%)* Women 0% *Age* T: 68.0, I: 68.2, C: 67.7 *Cancer type* PC *Stage* I to IV *Treatment* ADT | *Adherence rate* T: 78,3% *Univariable analysis* ↑ Exercise stage of change (r=0.31; *P=*0.004)**, ↑intention (r=0.30; *P=*0.006)**, ↓age (r= -0.29; *P=*0.009)**, ↑QoL (r=0.25, *P=*0.024)*, ↑fatigue (r=0.24; *P=*0.028)*, ↑subjective norm (r=0.23, *P=*0.037)*, ↑leg-press (r=0.23; *P=*0.041)*, ↑PBC (r=0.22; *P=*0.046)*, ↓type of ADT (r=-0.21; *P=*0.064), ↓cancer stage (*r*=-0.22; *P=0*.067) | | | *Multivariable analysis (HMRA)* ↑Exercise stage of change (β=0.26; *P=*0.013)*,  ↓age (β=-0.22; *P=*0.037)*,  ↑intention (β=0.19; *P=*0.073) *R^2^*20.4% | |  |
| **During treatment,** *home-based exercise intervention* | | | | | | | | |  |
| Shang, 2012, USA, no trial name [49] | Two- armed RCT | | *Population* (*n*) T: *n*= 126, I: *n*=68, C: *n*=58 *Gender (%)* Women, T: | *Adherence rate* I: 67,7%, C: 87.9% *Bivariable analysis*  ↑Exercise history (β=0.22; *P=*0.07), ↑physical fitness (β=0.19; *P=*0.10), ↓pre-treatment fatigue (β=-0.26; *P=*0.03)*, ↓mid-treatment mood disturbance (β=-0.21; *P=*0.09), | | | *Multivariable analysis (HPRA)* ↑Physical fitness (β=0.51; *P<*0.01)**, ↑exercise history (β=0.01; *P<*0.01)**, ↓pre-treatment fatigue (β=-0.93; *P<*0.01)**, ↓mid-treatment mood disturbance (β=-0.27; *P<*0.01)**, ↓married (β=-3.56; *P<*0.05)* | |  |
| **Supplementary Table 3.** *Continued* | | | | | |  | | |  |
| Author, year, country, trial name | Design | | Population ((*n*), gender, age (*±)*, cancer type, stage, treatment) | Results 1 (adherence rate, univariable analysis (P)) | | | Results 2 (multivariable analysis (*P*), R^2^) | |  |
|  |  | | 38.9%, I: 39.7%, C: 37.9% *Age* T: 60.2, I: 59.8, C: 60.6 *Cancer type* BC, CRC, PC, others *Stage* 0 to III *Treatment* RT, CT, COT, BT | ↓cancer type (t=-1.91; *P=*0.06), and ↓married (t= -2.30; *P=*0.02)***** | | | *R^2^*  75% | |  |
| **After treatment,** *center-based or a combined center- and home-based exercise intervention* | | | | | | | | |  |
| McNeely, 2012, Canada, no trial name [50] | Two- armed RCT | | *Population* (*n*) T: *n*=52, I: *n*=27, C: *n*=25 *Gender (%)* Women, T: 29%, I: 32%, C: 26% *Age* T: 52, I: 57, C: 53 *Cancer type* HNC *Stage* 0 to IV *Treatment* S, RT, CT | *Adherence rate* T: 91%, I: 95%, C: 87% *Univariable analysis (PCC)* ↑Education (r=0.277; *P=*0.047)*, ↑annual income (r=0.263; *P=*0.059), ↓disability status (r=-0.263; *P=*0.063), ↓type of neck dissection (r=-0.321; *P=*0.020)*, ↓alcohol consumption (r=-0.242; *P=*0.001)**, ↑QoL (r=0.307; *P=*0.027)*, ↓fatigue and anemia (r=0.234; *P=*0.096), ↓depression (r=-0.342; *P=*0.013)*, ↓anxiety (r=-0.303; *P=*0.029)* | | | *Multivariable analysis* ↓Extensive surgery (β=-0.361; *P=*0.007)**, ↓alcohol consumption (β=-0.298; *P=*0.031)*, ↑education (β=0.234; *P=*0.068), ↑annual income (β =0.100; *P=*0.465), ↓employment status (β=-0.156; *P=*0.282), ↑QoL (β=0.186; *P=*0.396), ↓fatigue and anemia (β=0.106; *P=*0.665), ↓ depression (β=-0.263; *P=*0.196), ↓anxiety (β=-0.063; *P=*0.715) *R^2^* 63% | |  |
| McGuire, 2011, USA, no trial name [51] | Two-armed RCT | | *Population* (*n*) T: *n*=233, I: *n*=120, C: *n*=113 *Gender (%)* Women 100% *Age* I: 58.7  *Cancer type* Postmenopausal BC survivors *Stage* 0 to II *Treatment* S, RT, CT | *Adherence rate* I: 61.89%,  *Bivariable analysis* ↑Previous adherence (r=0.60; *P=* 0.001)**, ↓married (t=-1.8; *P=* 0.074), ↓co-morbidities (r=-0.18; *P=*0.061), ↓BMI (r=-0.20; *P=*0.039)* | | | *Multivariable analysis (HMRA)* ↑Previous adherence (β=0.31; *P<*0.001)**, ↓married (β=0.10; *P=*0.032)*, ↑family support (β=-0.27; *P<*0.001)**, ↑feedback by trainers (β=0.40; *P<*0.001)**, ↑knowledge and skills of exercise (β=-0.14; *P=*0.032)* *R^2^ / Adjusted R^2^* 24.8%, 22.9% | |  |
| **After treatment,** *home-based exercise intervention* | | | | | |  | | |  |
| Kampshoff, 2016, The Netherlands, REACT study [52] | Three-armed RCT | | *Population (n)* T*: n*= 277, (*high intensity group)*:*n*= 139, (*low to moderate intensity group*): *n*= 138  *Gender (%)* Women 80% *Age*  T: 53.5, (*high intensity group)*: 54, (*low to moderate intensity group*): 53 *Cancer type* BC, CRC, PC, OC, CC, TC, LY *Stage* local/advanced  *Treatment* RT, CT, IT, COT, HT, S | *Adherence rate* (high intensity and low to moderate intensity group respectively)  *High session attendance:* 76% and 67%. *High compliance with RET:* 69% and 67%. *High compliance with AET:* 47% and 42%. *Univariable analysis - High session attendance*  *(high intensity group):* ↓psychological distress (*OR*: 0.93)*, ↑attitude (*OR*: 1.03)*, ↓perceived barriers (*OR*: 0.96)*, ↑self-efficacy (*OR*: 1.06)*, ↑exercise stage of change (*OR*: 2.96)* *- High compliance with RET*  *(high intensity group):* ↓psychological distress (*OR*: 0.87)*, ↑attitude (*OR*: 1.02)*, ↓perceived barriers (*OR*: 0.97)*  *(low to moderate intensity group):* ↓employment status (*OR*: 0.43)*, ↑being a non-smoker (*OR*: 0.18)*, ↑cancer types other than breast cancer (*OR*: 2.71)* *- High compliance with AET*  *(high intensity group):* ↓psychological distress (*OR*: 0.91)*, ↑attitude (*OR*: 1.02)*, ↑self-efficacy (*OR*: 1.05)*  *(low to moderate intensity group):* ↓education (*OR*: 0.45)*, ↑cancer types other than breast cancer (*OR*: 2.66)* | | | *Multivariable analysis (logistic regression) - High session attendance*  *(high intensity group):* ↑self-efficacy (*OR*: 1.06)* *- High compliance with RET*  *(high intensity group):* ↓psychological distress (*OR*: 0.87)*  *(low to moderate intensity group):* ↑being a non-smoker (*OR*: 0.16)*, ↑BMI (*OR*: 1.11)*, ↑cancer types other than breast cancer (*OR*: 3.25)* *- High compliance with AET*  *(high intensity group):* ↑self-efficacy (*OR*: 1.05)*  *(low to moderate intensity group):* ↑BMI (*OR*: 1.11)*, ↑cancer types other than breast cancer (*OR*: 3.25)*  *AUC* *- High session attendance*  *(high intensity group):* 0.75  *- High compliance with RET*  *(high intensity group):* 0.69  *(low to moderate intensity group):* 0.67  *- High compliance with AET*  *(high intensity group):* 0.68  *(low to moderate intensity group):* 0.69 | |  |
| Latka, 2009, USA, YALE Exercise and Survivorship Study [53] | Two-armed RCT | | *Population* (*n*) T: *n*= 75, I: *n*=37, C: *n*=38 *Gender (%)* Women 100% *Age* T: 55.8, I: 56.5, C: 55.1 *Cancer type* BC survivors  *Stage* 0 to IIIA | *Adherence rate* 81% *Univariable analysis (PCC)* ↓BMI (*P=*0.0103)*, ↓waist circumference (*P=*0.0326)*, ↑physical exercise 6 months prior to baseline (*P=*0.0207)*, ↑TTM stage of change (*P=*0.0055)**, ↓FACT-B Breast Cancer subscale score (*P=*0.0077)** | | | *Multivariable analysis* ↑exercise motivation (*P=*0.0306)*, ↓BMI (*P=*0.0424)* *R^2^*  N/a | |  |
| **Supplementary Table 3.** *Continued* | | | | | |  | | |  |
| Author, year, country, trial name | Design | | Population ((*n*), gender, age (*±)*, cancer type, stage, treatment) | | Results 1 (adherence rate, univariable analysis (P)) | | Results 2 (multivariable analysis (*P*), R^2^) | |  |
|  |  | | *Treatment* RT, CT, COT, HT, S | |  | |  | |  |
| Pinto, 2009, USA, MF trial [54] | Two-armed RCT | | *Population* (*n*) T: *n*=86, I: *n*=43, C: *n*=43 *Gender (%)* Women 100% *Age* T: 53.1, I: 53.4, C: 52.9 *Cancer type* BC survivors *Stage* 0 to II *Treatment* RT, CT, HT, S | | *Adherence rate* 69.8% *Univariable analysis* N/a | | *Multivariable analysis (GEE) - ± min of weekly physical exercise:* ↑Baseline self-efficacy (β=19.46; *P=*0.0004)**,  *- ± pedometer steps weekly:* ↑Baseline self-efficacy (β=2636.91; *P=*0.0006)**, ↑baseline physical exercise (β=1722.44; *P=*0.04)* *- ± achieving weekly goals:* ↑Baseline self-efficacy (β=0.48; *P=*0.0308)* *R^2^* N/a | |  |
| **During and after treatment,** *center-based or a combined center- and home-based exercise intervention* | | | | | | | | |  |
| Kuehl, 2016, Germany, PETRA study [55] | Two-armed RCT | *Population (n)* T: *n=* 153, I: *n=* 76, C: *n*=77 *Gender (%)* Women, T: 32.7%, I: 31.6%, C: 33.8% *Age* T: 53.8, I: 53.4, C: 54.2 *Cancer type* AML, ALL, LY/ CLL, MDS, CML/MPS, MM, other *Stage* Stage I to III CT, RT, allo-HCT | | | *Adherence rate* 66 to 78% *Univariable analysis (PCC) Inpatient stay* ↓physical fatigue (β=-0.04; *P=*0.04)*, ↑sport activity in adolescence (*competitive* β=0.17 and *any sports* β=0.40; *P=*0.024)*, ↑cognitive function (β= 0.01; *P=*0.036)*; ↓general fatigue (β=-0.03; *P=*0.040)* *Discharge ≤ day 100* ↑being married (β=0.08; *P=*0.030)*, ↑sub-maximal endurance capacity (β=0.01; *P=*0.002)*, ↓fatigue (β=-0.01; *P=*0.030)*, ↑emotional functioning (β=0.01; *P=*0.021)*, ↑social functioning ((β=0.01; *P=*0.031)* *Discharge > day 100* ↓inactive prior allo-HCT (β=-0.42; *P=*0.016)*, ↑sub-maximal endurance capacity (β=0.01; *P=*0.013)*, ↑adherence after discharge (β=0.01; *P=*0.001)** | | | *Multivariable analysis Inpatient stay* ↓physical fatigue (β= -0.04; *P=*0.04)*, ↑having children at home (β=-0.34; *P=*0.049)* *Discharge ≤ day 100* ↑sub-maximal endurance capacity (β=0.01; *P=*0.003)**, ↑emotional functioning (β=0.01; *P=*0.189), *Discharge > day 100* ↓inactive prior allo-HCT (β=-0.33; *P=*0.010)*, ↑adherence after discharge (β=0.01; *P=*0.001)** *R^2^**Inpatient stay* 16.0% *Discharge ≤ day 100* 21.2% *Discharge > day 100* 55.4% | |
| Craike, 2016, Australia, ENGAGE study [56] | Two-armed RCT | *Population* (*n*) T: *n*= 147, I: *n*=54, C: *n*=93 *Gender (%)* Women 0% *Age* T: 65.6, I: 66.9, C: 64.7 *Cancer type* PC *Stage* I to III *Treatment* S, ADT, RT | | | *Adherence rate* 80.3% *Univariable analysis* ↑ Role functioning (r=0.37; *P=*0.01)**, ↑sexual activity (r=0.25; *P=*0.06)*, ↓fatigue (r= -0.26; *P=*0.06), ↑hormonal symptoms (r=-0.31, *P=*0.03)*, ↑education (t=-2.65, *P=*0.01)** | | | *Multivariable analysis (GEE)* ↑Role functioning (β=3.09; *P=*0.019)*, ↑hormonal symptoms (β=-0.483; *P=*0.054) *R^2^* N/a | |
| Courneya, 2010, Canada, HELP trial [57] | Two-armed RCT | *Population* (*n*) T: *n*=122, I: *n*=60, C: *n*=62 *Gender (%)* Women, T: 41.0%, I: 38.3%, C: 43.5%  *Age* T: 53.2, I: 52.8, C: 53.8 *Cancer type* LY *Stage* 0 to IV *Treatment* RT, CT | | | *Adherence rate* 77.8% *Univariable analysis (PCC)* ↑Max planned cycles completed (r=0.42; *P=*0.026)*, ↑age (r=0.38; *P=*0.003)**, ↑previously regular exercisers or completely sedentary (r=-0.32; *P=*0.014)*, ↑no disease or disease stage IV (r=0.29; *P=*0.026)*, ↓BMI (r=-0.27; *P=*0.039)*, ↓previous treatments (r=0.24; *P=*0.060), ↓depression (r=-0.23; *P=*0.072), ↓smoking (r=-0.22; *P=*0.085) | | | *Multivariable analysis* ↑Age (β=0.29; *P=*0.016)*, ↑previously regular exercisers or completely sedentary (β=-0.27; *P=*0.024)*, ↓previous treatments (β=0.22; *P=*0.053), ↓BMI (β=-0.21; *P=*0.076), ↓smoking (β=-0.19; *P=*0.092) *R^2^*39.6% | |
| **During and after treatment,** *home-based exercise intervention* | | | | | | | | |  |
| Courneya, 2004b, Canada, CAN-HOPE trial [58] | Two-armed RCT | *Population* (*n*) T: *n*=93, I: *n*=62, C: *n*=31 *Gender (%)* Women, T: 41.9%, I: 45.2%, C: 35.5% *Age* T: 60.3, I: 59.9, C: 61.1 *Cancer type* CRC | | | *Adherence rate* 75.8% *Univariable analysis (PCC)* ↑Exercise stage of change (r=0.43; *P<*0.001)**, ↓tumor stage (r=-0.36; *P=*0.004)**, ↓treatment protocol(r=-0.37; *P=*0.003)**, ↓employment status (r=-0.35; *P=*0.005)**, ↓CT (r=-0.33; *P=*0.009)**, ↓RT (r=-0.28; *P=*0.028)*, ↑PBC (r=0.26; *P=*0.039)*, ↓age (r=0.26; *P=*0.044)*, ↑intention (r=0.22; *P=*0.080) | | | *Multivariable analysis (HMRA)* ↑Exercise motivation (β=0.35; *P=*0.001)**, ↓employment status (β=-0.28; *P=*0.010)**, ↓treatment protocol (β=-0.26; *P=*0.018)*, ↑PBC (β=0.20; *P=*0.055) *R^2^*39.6% | |
| **Supplementary Table 3.** *Continued* | | | | | |  | | |  |
| Author, year, country, trial name | Design | | Population ((*n*), gender, age (*±)*, cancer type, stage, treatment) | Results 1 (adherence rate, univariable analysis (P)) | | | Results 2 (multivariable analysis (*P*), R^2^) | |  |
|  |  | *Stage* Stage I to IV *Treatment* S, RT, CT, COT | | |  | | |  | |
| Courneya, 2002, Canada, GROUP-HOPE trial [59] | Two-armed RCT | *Population* (n) T: *n*=96, I: *n*=51, C: *n*=45 *Gender (%)* Women, T: 84.4%, I: 84.4%, C: 86.7% *Age* T: 51.6, I: 52.5, C: 50.5 *Cancer type* BC, CRC, LY, others *Stage* I to IV *Treatment* S, RT, CT, COT | | | *Adherence rate* 84.3% *Univariable analysis (PCC)* ↑Exercise history (r=0.50; *P<*0.001)**, ↑assignment to experimental condition (r=0.43; *P=*0.001)**, ↑gender (men) (r=0.38; *P<*0.001)**, ↑intention (r=0.31; *P=*0.002)**, ↑attitude (r=0.27; *P=*0.008)*, ↑treadmill time (r=0.26; *P=*0.012)*, ↑PBC (r=0.24; *P=*0.021)*, control beliefs (r=0.23; *P=*0.025)* | | | *Multivariable analysis (HMRA)* ↑Exercise history (β=0.36; *P<*0.01)**, ↑assignment to experimental condition (β=0.34; *P<*0.01)**, ↑gender (men) (β=-0.30; *P<*0.01)**, ↑exercise motivation (β=0.14; *P=*0.080)* *R^2^*50.6% | |
| *Statistical significant (*P<*0.05); **highly statistical significant (*P*≤0.01); ↑, higher/advanced results in better adherence; ↓, lower/less results in better adherence; *R^2^*, explained variance in exercise intervention adherence; AUC, area under the curve *Abbreviations*: T, total; I, intervention group; C, control group; RCT, randomized controlled trial; BC, breast cancer; CRC, colorectal cancer; PC, prostate cancer; LY, lymphoma; HNC, head and neck cancer; OC, ovarian cancer; CC, cervix cancer; TC, testis cancer; AML, acute myeloid leukemia; ALL, acute lymphoblastic leukemia; CLL, chronic lymphocytic leukemia; MDS, myelodysplastic syndrome; CML, chronic myeloid leukemia; MPS, myeloproliferatory syndrome; MM, Multiple myeloma; CT, chemotherapy; RT, radiation therapy; BT, brachy therapy; COT, combined therapy; HT, hormone therapy; S, surgery; ADT, androgen deprivation therapy; FEC-D, 5-fluorouracil, epirubicin, cyclophosphamide, docetaxel; wk, week; min, minutes; n/a, not available; BMI, body mass index; TTM, transtheoretical model; FACT-B, functional assessment of cancer therapy questionnaire for breast cancer; HMRA, hierarchical multiple regression analysis; OLHML, ordinal logistic hierarchical linear modeling; HPRA, hierarchical poisson regression analysis; GLM, generalized linear models; GEE, generalized estimated equations; PCC, Pearson correlation coefficient; OR, odds ratio; PBC, perceived behavioral control; AET, aerobic exercise training; RET, resistance exercise training; STAN, standard dose aerobic exercise training group; HIGH, high dose aerobic exercise training group; COMB, combined aerobic and resistance exercise training group | | | | | | | | |  |
